# Protective Mechanism of *Fagopyrum esculentum* Moench. Bee Pollen EtOH Extract Against Type II Diabetes in a High-Fat Diet/Streptozocin-Induced C57BL/6J Mice

**DOI:** 10.3389/fnut.2022.925351

**Published:** 2022-06-30

**Authors:** Jinjin Zhang, Wei Cao, Haoan Zhao, Sen Guo, Qian Wang, Ni Cheng, Naisheng Bai

**Affiliations:** ^1^College of Food Science and Technology, Northwest University, Xi'an, China; ^2^Bee Product Research Center of Shaanxi, Xi'an, China; ^3^Shaanxi Institute for Food and Drug Control, Xi'an, China

**Keywords:** polyphenols, type II diabetic mellitus, PI3K/AKT signaling pathway, bee pollen, high fat diet

## Abstract

Bee pollen is known as a natural nutrient storehouse and plays a key role in many biological processes. Based on the preliminary separation, identification, and characterization of the main active components of *Fagopyrum esculentum* Moench. bee pollen (FBP), the protective effects of *F. esculentum* bee pollen extract (FBPE) on high-fat-diet (HFD) and streptozocin (STZ) induced type II diabetes mellitus (T2DM) was evaluated in this study. The results revealed that FBPE contains 10 active compounds mainly including luteolin (9.46 g/kg), resveratrol (5.25 g/kg), kaemferol (3.67 g/kg), etc. The animal experiment results showed that FBPE could improve HFD-STZ induced T2DM mice. Moreover, the underlying mechanism of the above results could be: (i) FBPE could reduce the inflammation related to phosphatidylinositol 3-kinase/protein kinase B (PI3K/AKT) signaling pathway, and (ii) the gut microbiota remodeling. The results of correlation analysis showed *Candidatus Arthromitus* and *SMB53* indicated positive correlations to tumor necrosis factor-α (TNF-α); *Coprococcus, Ruminocossus*, and *Odoribacteraceae* reported negative correlations to transforming growth factor-β (TGF-β). That FBPE has an outstanding ability to improve T2DM and could be used as a kind of potential functional food for the prevention of T2DM.

## Introduction

Diabetes mellitus (DM) is a chronic disease caused by defective insulin action, defective insulin secretion, or both, usually manifesting as hyperglycemia (1). And long-term elevated blood glucose level is prone to complications such as neuropathy, kidney disease, heart disease, retinopathy, etc. Diabetes is divided into type I diabetes mellitus (T1DM) and type II diabetes mellitus (T2DM), while the latter is the most prevalent form, and its progression includes the development of insulin resistance, impaired glucose tolerance, and eventual pancreatic β-cell dysfunction (2). And accompanied by aging, obesity, and the popularity of high-calorie food, high-fat food, and fast food, T2DM has become an incidence-rising disease in the world (3). So, if it can't be controlled in time and effectively, it will seriously endanger health. Traditional medicine for T2DM treatment mainly includes insulin, biguanide, sulfonylureas, meglitinides, thiazolidinediones, and so on (4). Studies proved that these drugs can keep blood sugar levels from rising effectively, but long-term usage could exhibit certain side effects (5, 6). Therefore, it is necessary to find new potential natural products that can prevent T2DM.

Bee pollen is a product of male reproductive cells collected by the honeybee *Apis mellifera* from flower stamens of gymnosperm and angiosperm (7). Many studies have reported that it contains more than 250 kinds of biologically active substances such as polysaccharides, essential amino acids, phenolic acids, unsaturated fatty acids, and flavonoids (8). Concerning the research on bee pollen, they have focused on anti-diabetic activities as well as other biological functions including antioxidation, antitumor, improving cardiovascular and cerebrovascular diseases, treating prostate diseases, and so on (9). And recent research showed that polysaccharides in bee pollen can regulate gut microbes (10). FBP is a pollen ball formed by honeybee collecting pollen of *Fagopyrum esculentum* Moench. and add saliva and honey. It is wildly distributed in China and used as a functional food for several decades. Our research group previously isolated 16 compounds from FBP and proved kaempferol exhibited high-glucosidase inhibitory activity (IC_50_: 80.35 μg/mL) (11). Yet, there is only a little research has been published about the bio-activities of FBP, especially anti-diabetes activity in T2DM mice.

The phosphatidylinositol 3-kinase/protein kinase B (PI3K/AKT) signaling pathway regulates metabolism in normal physiology and morbid conditions, and diseases such as obesity and diabetes are related to it (12). It is the main downstream pathway of insulin action and the most important one among the various pathways in which insulin regulates glucose and lipid metabolism in diabetes. In T2DM, this pathway is blocked, insulin secretion and β-cell function are reduced, and reactivation of this pathway can alleviate this condition. Moreover, a variety of drugs have been reported to exert hypoglycemic effects via PI3K/AKT signaling pathway (13–15). Therefore, we investigated the effect of FBPE on the pancreatic PI3K/AKT signaling pathway, which will be beneficial to study the hypoglycemic mechanism of FBPE in T2DM mice.

In this article, we identified the phenolic composition and investigated the palliative effects of FBPE on T2DM in C57BL/6J mice. The potential hypoglycemic mechanism of FBPE via the PI3K/AKT signal pathway on the mRNA level was also evaluated. More importantly, the regulatory effect of FBPE on HFD-STZ induced gut microbiota disturbance on T2DM was studied. These would provide theoretical support for studying the biological activity of FBPE and developing related functional products.

## Materials and Methods

### Materials and Chemicals

**Figure 1:**
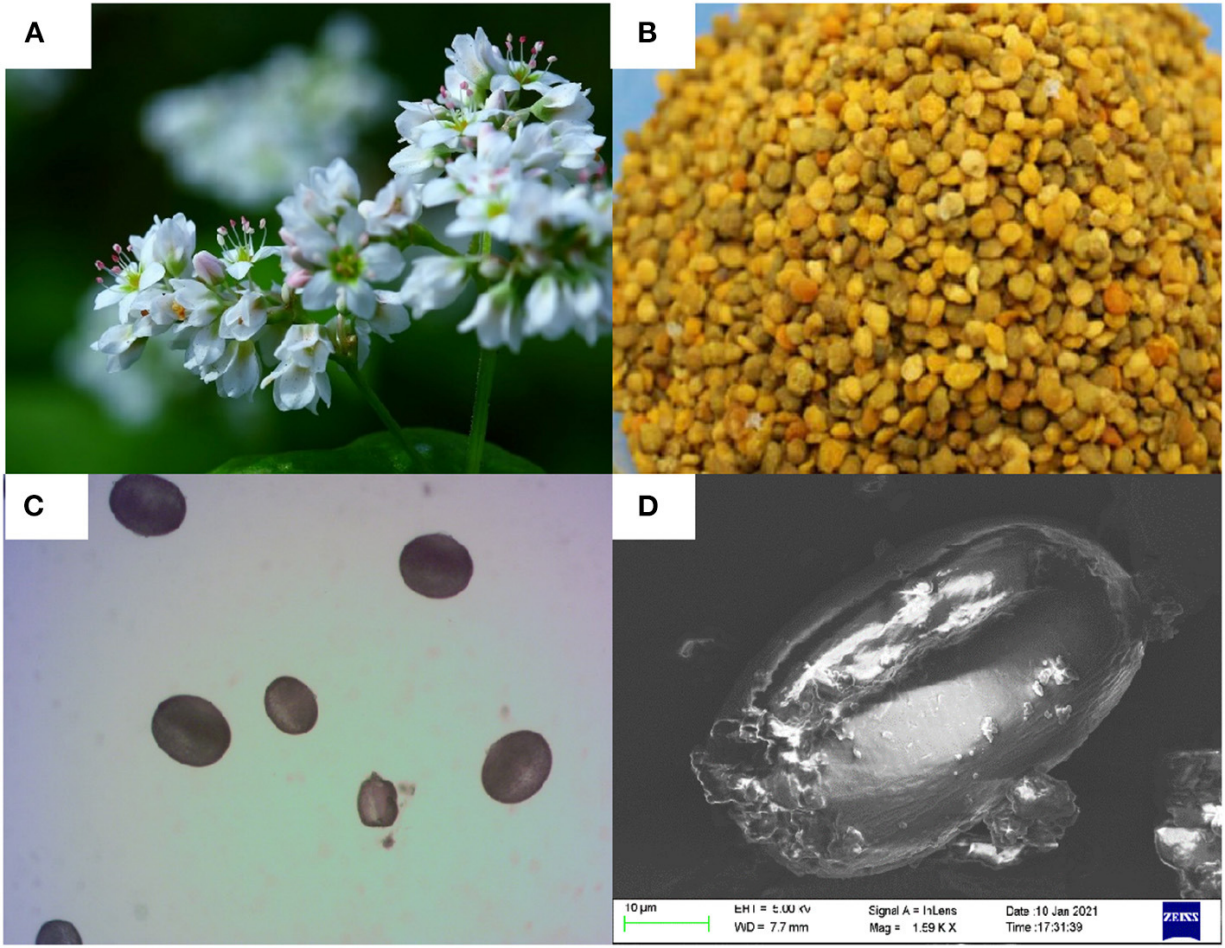
**(A)** Flower, **(B)** bee pollen, the melissopalynological analysis by, **(C)** a bright-field microscope (200×magnification), and **(D)** scanning electron micrographs of *Fagopyrum esculentum* Moench.

The FBP was collected from Jingbian, Shannxi, China in 2018 and stored at 4°C. Grind the pollen particles into powder, and the melissopalynological was performed by a bright-field microscope (Olympus, Tokyo) at 200 magnification (Figure 1C). Scanning electron micrographs of FBP (Figure 1D) were observed by a Hitachi S-750 SEM system (Hitachi Company, Japan). All other chemicals reagents used were analytical grade.

### Preparation of EtOH Extract of *F. esculentum* Bee Pollen

The collected FBP (5 kg) was percolated at room temperature with 90% EtOH, the sample was completely immersed in EtOH and mixed well. The mixture was stirred every 3 h, fully filtered out the filtrate after 2 days, repeated 3 times, and combined the filtrates and concentrated under vacuum to yield EtOH extract (FBPE, 1.97 kg).

### Estimation of TPC, TFC and Antioxidant Capacity *in vitro*

The estimation of total phenolic content (TPC) and total flavonoids content (TFC) were performed according to the methods described by Cheng et al. (16) and Wang et al. (17), respectively, for FBPE. Briefly described as follows: For TPC determination: Prepare a FBPE solution with a concentration of 2 mg/mL. Transfer 1 mL of this FBPE solution into a test tube, 1 mL of Folin-Ciocalteu reagent, and 5 mL of 1 mol/L sodium carbonate solution followed. Finally, add 3 mL of ultra-pure water into the tube to ensure the total volume of the final mixture is 10 mL. The absorbance of the mixture was measured at 760 nm after being well-mixed and incubated in the dark for 60 min. The TPC was expressed as the gallic acid equivalents per gram FBPE (mg GAE/g). For TFC determination: Prepare a FBPE solution with a concentration of 0.05 g/mL. Transfer 1 mL of this solution into a tube and 0.4 mL of 5% sodium nitrite solution was added. Let stand for 6 min, 0.4 mL of 10% aluminum nitrate was added. And 4 mL of 4% sodium hydroxide was added after 6 min and the total was made up to 10 mL with methanol. The solution was mixed well again and the absorbance was measured against a blank at 510 nm 15 min later. The TFC was expressed as the rutin equivalents per gram FBPE (mg RE/g). And the DPPH radical scavenging activity and ferrous ion-chelating activity were determined according to methods described by Cheng et al. (16).

### NMR Analysis of FBPE

The FBPE was separated by column chromatography and identified by thin-layer chromatography (TLC), high performance liquid chromatography (HPLC), mass spectrometry (MS), ultraviolet spectrum (UV), hydrogen nuclear magnetic resonance spectrum (^1^H-NMR), carbon nuclear magnetic resonance spectrum (^13^C-NMR) and other techniques (11). A total of 10 compounds were obtained and 8 flavonoids were quantified by HPLC.

### HPLC Analysis of FBPE

**Figure 2:**
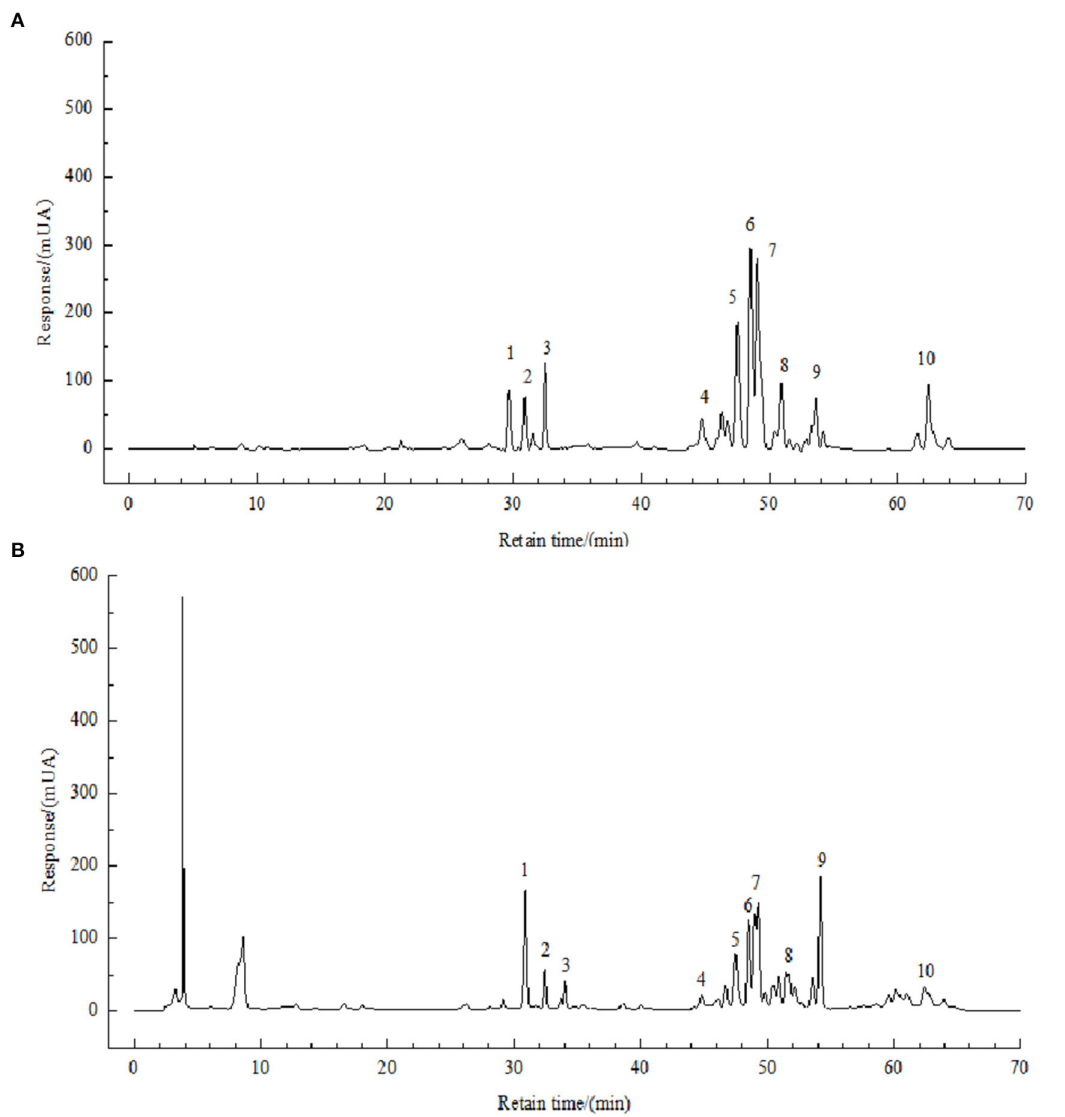
HPLC chromatograms of solution of standards **(A)** and FBPE **(B)** at 210 nm. Peaks: chlorogenic acid (1), caffeic acid (2), resveratrol (3), catechin (4), luteolin (5), rutin (6), kaempferol (7) and quercetin (8).

In a previous study, we developed an HPLC method for the identification of polyphenols in FBP, and the method was described in Li et al.'s (11) paper. The chromatograms were shown in Figure 2.

### Animals and Experimental Design

A total of 48 male C57BL/6J mice (20 ± 2 g) were purchased from Xi'an Jiaotong University School of Medicine (laboratory animal production license number: SCXK (Shaan) 2017-003). All mice were housed in an environment with suitable temperature and humidity (24 ± 2°C, light/dark cycle of 12/12 hours) with free to eat and drink. All animal procedures were performed following the Guidelines for Care and Use of Laboratory Animals of Northwest University and experiments were approved by the Animal Ethics Committee of Northwest University (NWU-AWC-20190105M).

#### Established T2DM Model

All mice were acclimated for a week, followed by an HFD for 4 weeks, after a 12-h fast, all the mice have injected with STZ (55 mg/Kg, dissolve with freshly prepared 0.1mol/L citric acid buffer). Three days after STZ injection, fasting blood glucose (FBG) was tested, and the mice with an FBG value > 16.7 mmol/L were used for the subsequent experiments.

#### Experiment

The T2DM mice were randomly divided into 4 groups: (1) diabetic model group (DG, *n* = 12); (2) positive group (Metformin, MET, 150 mg/kg BW) (PG, *n* = 10); (3) low dose FBPE (1 g/kg BW) treatment group (LG, *n* = 10) and (4) high dose FBPE (6 g/kg BW) treatment group (HG, *n* = 10). The four group mice were given carboxymethylcellulose sodium solution (NaCMC, 0.5%), MET, or FBPE by intragastric administration and all the animals were continually fed with HFD.

### Sample Collection

After 8 weeks, blood samples were collected, then centrifuged at 2,500 r/min for 20 min after clotting for 2 h at room temperature to obtain serum. The colon content, epididymal adipose, pancreas, and liver were collected and preserved at −80°C.

### Biochemical Analysis

Serum concentrations of insulin, total cholesterol (TC), triglyceride (TG), low-density lipoprotein-cholesterol (LDL-C), high-density lipoprotein-cholesterol (HDL-C), cereal third transaminase (ALT), aspartate transaminase (AST), alkaline phosphatase (ALP), albumin, and the levels of hepatic glycogen, TC, TG, total protein (TP), superoxide dismutase (SOD), glutathione peroxidase (GSH-Px) and malondialdehyde (MDA) were determined using commercially available diagnostic kits according to the instructions (JianCheng, Nanjing, China). The homeostasis model assessment of insulin resistance (HOMA-IR), insulin sensitivity index (ISI), and homeostasis model assessment-β (HOMA-β) were calculated according to the following formulas, respectively (14, 18):


(1) $$HOMA-IR=\frac{FBG(mmol/L)\times FINS(mIU/L)}{22.5}$$

(2) $$ISI=\frac{1}{INS\times FBG}$$

(3) $$HOMA-\beta =\frac{20\times INS}{FBG-3.5}$$

### Histological Analysis

Histological analysis was performed by hematoxylin and eosin (H&E) staining. The specific operation steps were as follows: take a portion of the liver and epididymal adipose, fix it in 10% neutral formalin, embed it in paraffin wax, cut 5 μm thick sections, dewaxed, dehydrated, and stained with H&E.

### Real-Time Quantitative PCR Analysis

The pancreas tissue was used to measure gene expression of PI3K, AKT, interleukin-2 (IL-2), interleukin-2 (IL-6), TNF-α, and TGF-β. A real-time quantitative polymerase chain reaction (RQ-PCR) assay was according to Chen et al. (19). The primer sequences (Supplementary Table 1) of IL-6, IL-2, PI3K, AKT and Glyceraldehyde-3-phosphate dehydrogenase (GAPDH) were designed refer the method proposed by Liu et al. (20). The primer sequences of TNF-α and TGF-β were designed according to other two studies (21, 22). The purity of PCR products was assessed by melt curve analysis. GAPDH was amplified as an internal control. The mRNA expression was quantified using the comparative cycle threshold method (2^−ΔΔCT^) according to Livak and Schmittgen (23).

### Gut Microbiota Analysis

Total genomic DNA was extracted from colon contents using the Fast SPIN extraction kits (MP Biomedicals, Santa Ana, CA, USA). The quantity and quality of extracted DNA were measured using a NanoDrop ND-1000 spectrophotometer (Thermo Fisher Scientific, Waltham, MA, USA) and agarose gel electrophoresis, respectively. PCR amplification of the bacterial 16S rRNA genes V3-V4 hypervariable regions, the product was subjected to fluorescence quantification, the fluorescent reagent was Quant-iT PicoGreen dsDNA Assay Kit, and the quantitative instrument was a microplate reader (BioTek, FLx800). Sequencing was performed using Illumina's MiSeq platform (Personal Biotechnology Co., Ltd., Shanghai, China). Then the quantitative insights into microbial ecology (QIIME, v1.8.0) pipeline was employed for bioinformatics analysis (24). The operational taxonomy units (OTUs) of representative sequences at 97% similarity were used to calculate the diversity index. Non-metric multidimensional scaling (NMDS) examined the abundance and diversity of the OTUs.

### Statistics Analysis

The data were expressed as the mean ± standard deviation (SD). Differences between groups were assessed by one-way analysis of variance (ANOVA) followed by Tukey's multiple range test using SPSS 25.0 software. *P* < 0.05 was considered statistically significance.

## Results and Discussion

### TPC, TFC and Antioxidant Capacity *in vitro*

As shown in Supplementary Table 2, the TPC was determined as 18.59 ± 1.39 g GAE/kg, and TFC was 16.35 ± 0.09 g RE/kg, the results of antioxidant activity *in vitro* showed that the DPPH free radical scavenging ability was 11.01 mg/mL (IC_50_ value), and the ferrous ion-chelating activity was 32.84 ± 1.49 mg EDTA-2Na/g.

### Identification and Quantification of FBPE

Compounds 1–10 were identified as chlorogenic acid, caffeic acid, resveratrol, (-)-catechin, luteolin, rutin, kaempferol, tyrosol, quercetin, and octacosanol. The NMR data and spectrum were shown in the supporting information in the previous article published by our research group (11). On this basis, we briefly described as follows:

Compound 1 (chlorogenic acid) is a white powder, possessed the molecular formula C_16_H_18_O according to its HR-ESI-MS (m/z: 354.31). The ^1^H-NMR (400 MHz, methanol-d_4_) data revealed the existence of multiple-OH (δ_H_4.08 and δ_H_2.0), and ^13^C-NMR (101 MHz, methanol-d_4_) data revealed the existence of double bond (δc149.58 and δc115.19) and -COOH (δc177.03 and δc168.62). Compound 2 (caffeic acid) is a white powder, possessed the molecular formula C_9_H_8_O_4_ according to its HR-ESI-MS (m/z: 180.15). The ^1^H-NMR (400 MHz, DMSO-d6) data indicated the existence of -COOH (δ_H_12.16), the ^13^C-NMR (101 MHz, DMSO-d_6_) data indicated the existence of -C=O (δ_C_168.41). Compound 3 (resveratrol) is a brown solid having a molecular formula C_14_H_12_O_3_ established by its HR-ESI-MS (m/z: 354M^+^). In the ^1^H-NMR (400 MHz, DMSO-d_6_) spectrum, δ_H_6.93 and δ_H_6.81 were the hydrogen atoms of olefin, and the coupling constants 16.4 Hz suggest the trans-structure. δ_H_7.34 and δ_H_6.75 showed a set of AAXX spin systems, and δ_H_6.38 and δ_H_6.11 showed a set of AX_2_ spin systems. In addition, δ_H_9.24 and δ_H_9.60 in the low field indicated the signal peaks of three active hydroxyl groups. Compound 4 ((-)-catechin) is a yellow oil, the molecular formula was C_15_H_14_O_6_ established by its HR-ESI-MS (m/z: 290.27). In ^1^H-NMR (400 MHz, DMSO-d6) spectrum, the signals in high field regions δ_H_6.58, δ_H_6.67, and δ_H_6.71 indicated the existence of the typical ABX coupling system on the benzene ring in the structure, and δ_H_5.67 and δ_H_5.87 were two hydrogen proton signals of coupling between benzene rings. Moreover, δ_H_8.81, δ_H_8.85, δ_H_8.93 and δ_H_9.17 in the low field revealed the existence of four phenolic hydroxy hydrogen protons, the ^13^C-NMR (101 MHz, DMSO-d_6_) data showed the alkyl carbon signals (δ_C_28.2) and carbon signals that were connected to oxygen (δ_C_65.0, δ_C_80.8). Compound 5 (luteolin) is a yellow powder, which HR-ESI-MS was m/z: 290.27, and the molecular formula was speculated C_15_H_10_O_6_, suggesting 11 degrees of unsaturation. In ^1^H-NMR (400 MHz, DMSO-d_6_) spectrum, δ_H_12.8 was the signal of the -position hydroxyl proton of flavonoids, and the δ_H_7.43, δ_H_7.41 and δ_H_6.90 in the low field region constitute the ABX system, indicating that the B ring is 3', 4'-dioxy substituted, δ_H_6.46 and δ_H_6.20 were the signals of the typical 5, 7-dioxy substituted flavonoid A ring. Moreover, the ^13^C-NMR (101MHz, DMSO-d_6_) data revealed the existence of carbonyl (δ_C_182.12). Compound 6 (rutin) is a yellow powder and possessed the molecular formula C_27_H_30_O_16_ according to its HR-ESI-MS (m/z: 610.52). HPLC-DAD data showed there were large absorption peaks at 280 nm and 360 nm and this compound was presumed to be a flavonoid compound. In the ^1^H-NMR (400 MHz, DMSO-d_6_) spectrum, δ_H_12.61 was the signal of the hydroxyl protons at the 5' position of the flavonoid A ring, and δ_H_6.20 and δ_H_6.39 revealed the existence of the protons at 6 and 8 positions in the 5, 7- dioxy-substituted flavonoid A ring. Additionally, δ_H_7.54, δ_H_6.84, and δ_H_7.56 were the signals of the 2',5',6' protons of the B-ring of flavonoids, respectively. Ans δ_H_5.34 was the matrix sub-signal of the glucose terminal. The coupling constant J = 7.0Hz indicates that glucose is β-configuration. Moreover, δ_H_1.00 was rhamnose CH_3_ proton signal, the ^13^C-NMR spectrum showed 27 carbon signals, δ_C_178.00 and δ_C_16.48 revealed a carbonyl carbon and methyl carbon, respectively. In addition, δ_C_164.61-103.22 ware speculated to be the carbon signal of a benzene ring and a double bond in flavonoids in combination with ultraviolet and ^1^H-NMR profiles. Compound 7 (kaempferol) is an amorphous yellow powder, had a molecular formula C_15_H_10_O_6_ deduced by HR-ESI-MS (m/z: 286M^+^). In ^1^H-NMR (400 MHz, DMSO-d_6_) spectrum, δ_H_12.49, δ_H_10.80, δ_H_10.13 and δ_H_9.43 revealed the existence of phenolic hydroxyl and the ^13^C-NMR (101 MHz, DMSO-d_6_) data (δ_C_168.41) indicated the existence of carbonyl carbon. Compound 8 (tyrosol) is yielded as a white powder. The ^13^C-NMR (101 MHz, methanol-d_4_) indicated the existence of benzene ring (δ_C_130.6, 130.7, 1565, 130.8 and 116.0), the ^1^H-NMR (400 MHz, methanol-d_4_) data revealed the position of hydrogen (δ_H_7.31, 7.16, 4.08 and 3.02). Compound 9 (quercetin) is a yellow powder, had a molecular formula C_21_H_20_O_11_ deduced by its HR-ESI-MS (m/z: 286M^+^). The ^1^H-NMR (400 MHz, DMSO-d_6_) spectrum showed the hydrogen proton signal of aromatic protons and rhamnose. And ^13^C-NMR (100 MHz, DMSO-d_6_) data revealed the existence of methyl carbon (δ_C_18.01) and carbonyl carbon (δ_C_179.0). Compound 10 (octacosanol) is a colorless, odorless powder. It has no UV absorption in HPLC-DAD detection and the ^1^H-NMR (400 MHz, DMSO-d_6_) and ^13^C-NMR (101 MHz, DMSO-d_6_) data are displayed there were no double bond and no ring, and speculated that the structure is directly connected.

Then, the content of 8 phenolic compounds of them was analyzed. As shown in Supplementary Table 3, luteolin has the highest content of 9.46 g/kg, followed by resveratrol with 5.25 g/kg, chlorogenic acid, and rutin have the lowest content of 1.45 g/kg.

### Situation Affected by FBPE and MET

#### Effects on BW, FBG and Insulin

**Figure 3:**
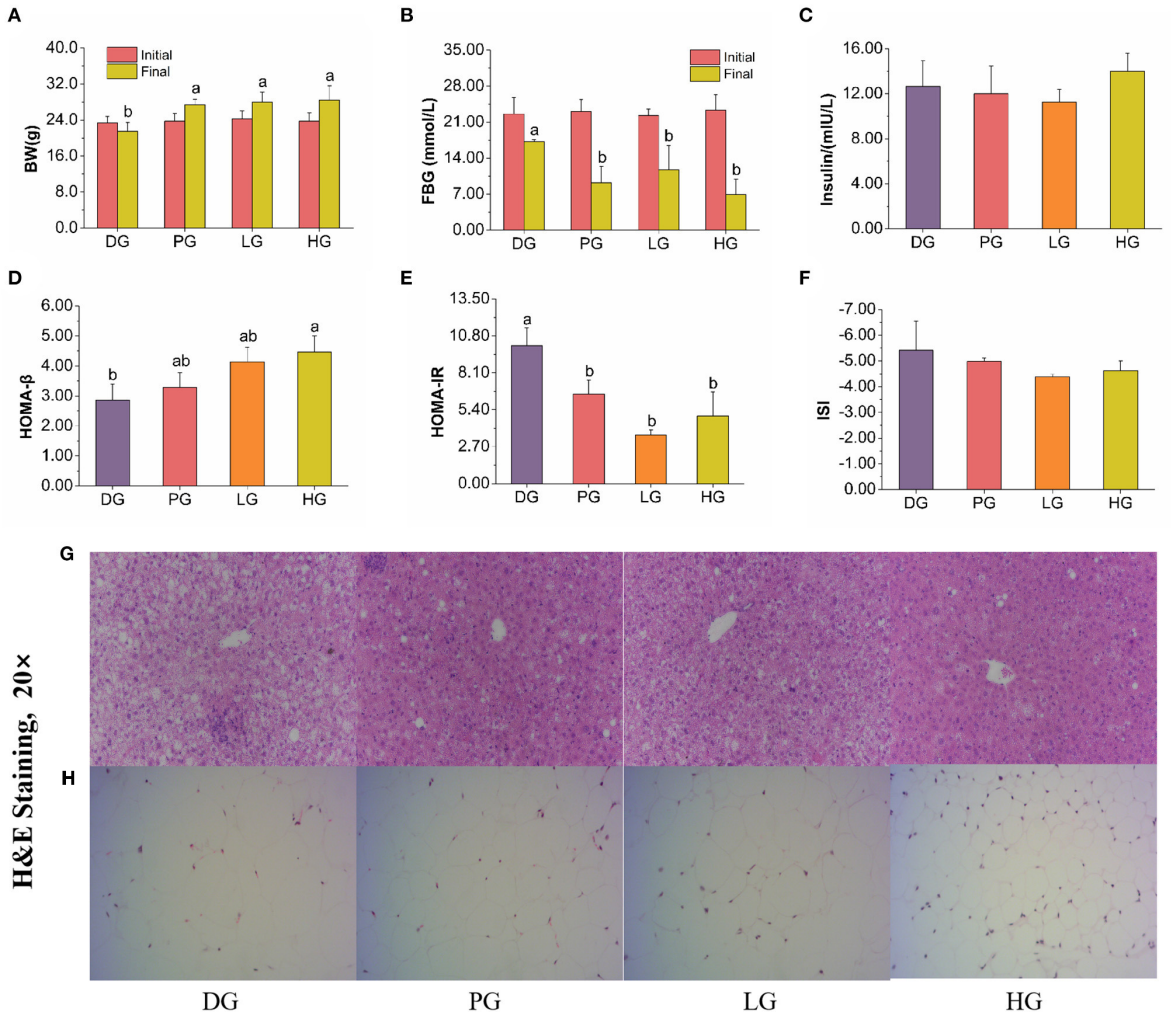
Effects of MET or FBPE on the development of T2DM in HFD-STZ-treated mice. **(A**–**F)** Changes in BW, FBG, insulin, HOMA-β, HOMA-IR, and ISI of T2DM mice after treatment with MET or FBPE, effects of FBPE or MET on the liver **(G)**, and epididymis fat **(H)** histology (H&E stain, 20×). Dates are presented as mean ± SD, (*n* = 10), different letters mean significantly different, *P* < 0.05.

STZ induced T2DM mice are often characterized by weight loss, blood sugar increase, and insulin resistance. In our study, FBPE for the treatment of T2DM provided favorable data on both FBG and BW compared with MET, a potent medicine in T2DM. BW and FBG were measured at initial and intragastrically 8 weeks, respectively. As shown in Figure 3, MET and FBPE administration could improve BW but decrease FBG. In addition, we found that MET and FBPE administration could markedly enhance insulin sensitivity and alleviate IR in comparison with the model group by calculating HOMA-IR, ISI, and HOMA-β. In Ma et al.'s (25) study, rambutan peels (*Nephelium lappaceum*) extract, which is rich in catechin and ellagic acid, has hypoglycemic effects. And in another report, the FBG inhibitory activity of *Pistacia lentiscus* was associated with gallic acid, catechin, and ellagic acid in its constituents (26). In our research, we identified 8 kinds of phenolics from FBPE by HPLC analysis. Thus, it was speculated that the anti-diabetes activity of FBPE may be related to the high content of flavonoids.

#### Effects of FBPE on HFD-STZ Induced T2DM in Serum Analysis

Long-term elevated blood glucose levels could lead to lipid metabolism disorder, usually expressed as increased TC, TG, LDL-C, and decreased HDL-C (27). These changes may induce atherosclerosis, coronary heart disease, stroke, pancreatitis, aggravated hepatitis, or other injuries (28). Growing evidence suggests that natural products have positive regulatory effects on lipid metabolism disorders (29–31). In serum biochemical analysis (Supplementary Table 4), FBPE could decrease TG, LDL-C, and increase HDL-C levels, demonstrating that FBPE or MET could prevent serum lipid accumulation.

As mentioned earlier, ALP is widely distributed in many organs of the human body (32), and elevated serum ALP level are frequently associated with liver diseases (33). Moreover, the serum AST and ALT activity also can reflect the degree of liver cell damage and necrosis sensitively (34). After FBPE administration, ALT and AST activity were decreased, but no significant effect on ALP activity was noticed. As expected, the results revealed that MET and high-dose FBPE administration could protect liver cells, and rescue liver injury in T2DM mice.

#### Effects of FBPE on HFD-STZ Induced T2DM in Liver Analysis

After determining the serum biochemical parameters, we evaluate the effects of FBPE on liver indices of T2DM mice. In liver biochemical analysis (Supplementary Table 4), only high-dose FBPE administration effectively reduced the TC level in T2DM mice, all PG, LG, and HG mice express lower TG levels after 8 weeks. These revealed that FBPE treatment could alleviate liver lipid accumulation. Insulin affects Glucose metabolism in the liver (35). When stimulated by insulin, hepatocytes convert glucose into glycogen to maintain glucose homeostasis (36). To a certain content, hepatic glycogen level can reflect insulin activity (37). In this paper, a significant increment of hepatic glycogen level in LG mice was noticed also, it could be speculated that FBPE treatment may effective in enhancing insulin sensitivity, which echoes the previous results.

Oxidant stress is a major mechanism for the progression of diabetes and could lead to cellular damage (38). The production of reactive oxygen species (ROS) in the body is increased in a hyperglycemic environment, which could decrease the antioxidant defense mechanisms and lead to vascular complications (28). Thus, this research further evaluated the protective effect of FBPE in T2DM mice by determining the endogenous antioxidant enzymes and liver lipid peroxide. In our experiment, high-dose FBPE treatment increased the SOD activity in T2DM mice after 8 weeks, and GSH-Px activity was significantly improved after treating with low-dose FBPE (Supplementary Table 4). SOD and GSH-Px are two endogenous antioxidant enzymes that can effectively relieve oxidative stress in *vivo*. The results indicated that FBPE administration could improve scavenging toxic ROS ability in T2DM mice. Besides, FBPE is rich in polyphenols and has shown strong antioxidant activity, which is speculated to be one of the possible mechanisms to protect the enzyme activity in diabetic mice. MDA, which reflected the degree of lipid peroxidation, is a by-product of lipid peroxidation, and obesity and diabetes patients are usually accompanied by an elevated MDA level in the liver (39). The results showed that FBPE could inhibit the increase of MDA content in a dose-dependent manner (Supplementary Table 4). This indicated that antioxidants contained in FBPE could inhibit hepatic lipid peroxidation in T2DM mice. These are consistent with a study about *Punica granatum Flower* polyphenols extracts in T2DM rats, which demonstrated that *Punica granatum Flower* can reduce lipid accumulation and alleviate oxidative stress in rats (40).

### Histopathological Analysis

In other reports, the hepatocytes of normal mice showed distinct cell borders, and the central veins with rounded nuclei surrounded by abundant cytoplasm (28). As shown in Figure 3G, the T2DM mice were observed obvious changes, including mussy hepatic cords, intercellular space increased, hepatocyte hypertrophy, cytoplasmic vacuolation, nucleus disappearance, and infiltration of inflammatory cells. Compared with DG mice, MET treatment attenuated pathological damage. In response to the administration of FBPE for 8 weeks, the hepatic lesions were ameliorated to different extents and demonstrated a dose-dependent protective effect in the T2DM mice. High-dose-FBPE effectively alleviated the symptoms of hepatocyte hypertrophy, hepatic lipid accumulation, and infiltration of inflammatory cells, indicating the superior ability of FBPE to ameliorate the progressive deterioration of hepatic lesions in T2DM. In addition, lipid accumulation and adipose distribution of epididymis fat were examined in the experimental mice. The adipocytes of normal mice always consist of lipid vacuoles surrounded compactly by a thin rim of cytoplasm (41). In our study, we observed adipocyte enlargement and intercellular connective tissue in DG mice. After MET administration, the adipocyte enlargement of T2DM mice was significantly restored and distinct pockets were observed. It was worth noting that the corresponding improvement effect was also observed in LG and HG mice, which showed a dose-dependent effect (Figure 3H).

### Effect of FBPE on Related Genes Expression

**Figure 4:**
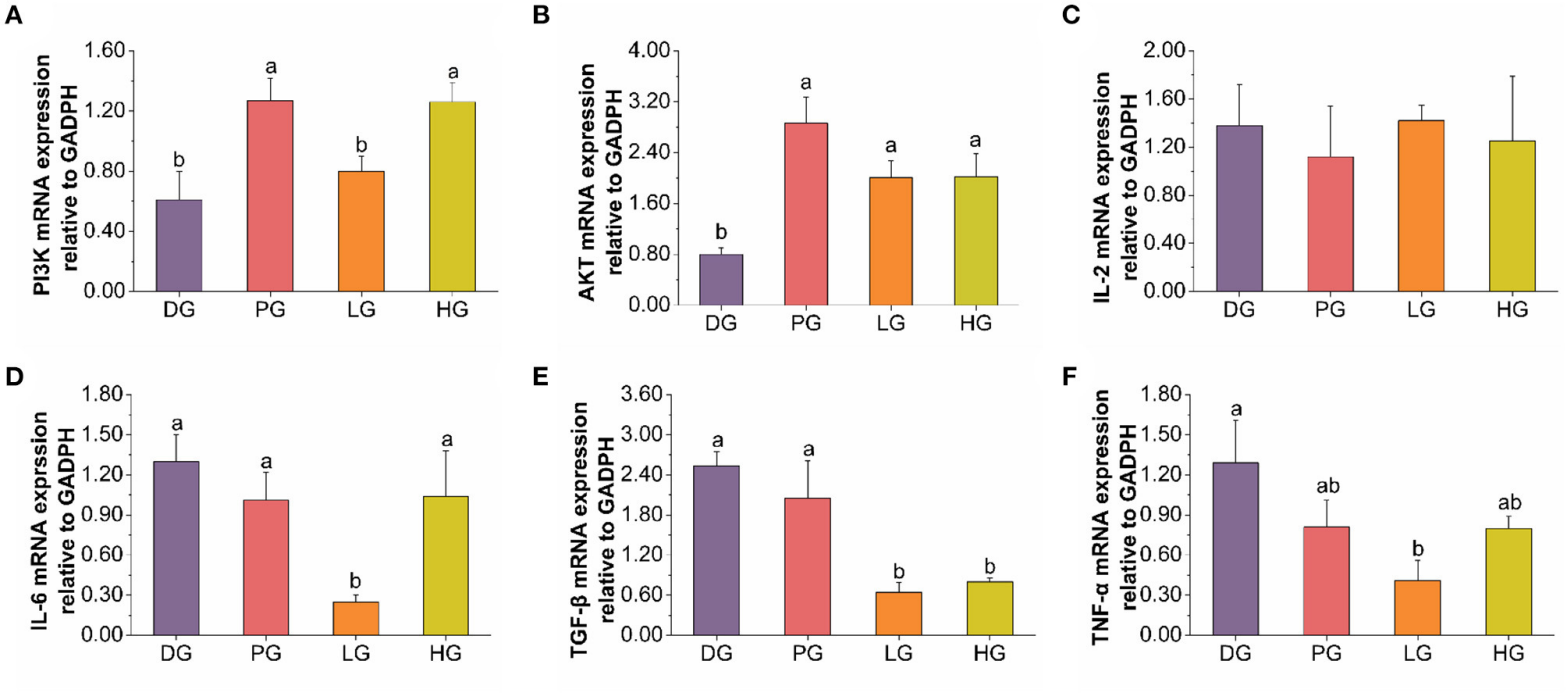
Effects of FBPE or MET on the release of inflammatory cytokines in T2DM mice. **(A–F)** mRNA expression of PI3K, AKT, and inflammatory cytokines IL-2, IL-6, TGF-β, and TNF-α in pancreas tissues, respectively. Dates are presented as mean ± SD, (*n* = 10), different letters mean significantly different, *P* < 0.05.

T2DM can cause low-grade chronic systemic inflammation, and enhance the production of IL-6, TNF-α, and TGF-β in response to HFD (42, 43). Both IL-6 and TNF-α can induce pancreatic β-cells apoptosis in diabetic patients, especially in STZ-induced diabetes (20). And pancreatic β cells in the pancreas are vital in insulin-producing and secreting to maintain glucose homeostasis (44). As shown in Figures 4C–F, the mRNA expression of TNF-α and TGF-β were down-regulated in low-dose FBPE administrated mice. And the high-dose FBPE down-regulated TGF-β mRNA expression but didn't improve that of TNF-α. Moreover, a down-regulated mRNA expression of IL-6 was observed in LG mice whereas didn't observe in MET and HG mice. Additionally, MET, low-dose FBPE, and high-dose FBPE treatment didn't improve the mRNA expression of IL-2 compared with DG mice after 8 weeks. These indicated that FBPE could relieve T2DM Induced inflammation to some extent. The PI3K/AKT signaling pathway is particularly important for β cell function (12), and it could promote insulin secretion from pancreatic β cells (45, 46). However, in T2DM, this pathway is blocked and β cell function is impaired (44). Thus, it is very important in blood glucose regulation. Additionally, numerous natural products have been confirmed to exert their hypoglycemic effects through the PI3K/AKT signaling pathway. Gao et al. (47) found that sea buckthorn fruit oil (SBFO) extract could promote the expression of PI3K while inhibiting the expression of glycogen synthesis kinase-3β (GSK-3β) which revealed that it could alleviate T2DM through the PI3K/AKT signaling pathway. And the anti-diabetic effect of *citrus pectin* (48), *Dendrobium officinale* polysaccharide (49), corn silk *(Maydis stigma)* polysaccharide (50), and tea polysaccharides (51) also had been proved associated with the activation of PI3K/AKT signaling pathway, respectively. So, to elucidate the hypoglycemic mechanism of FBPE in T2DM mice, we assessed the mRNA expression of PI3K and AKT in the pancreas. PI3K, which is important for insulin signaling transduction, is a target protein for insulin receptor substrates (52). AKT is one of the main effectors of this transduction process and is important in many physiopathological processes and cellular regulation (51). And it is activated by PI3K in response to growth factors, Ca^2+^ influx, and extracellular stressors such as oxidative stress (53). An increased mRNA expression of AKT and PI3K was observed in the pancreas of T2DM mice after supplementation with FBPE or MET in this study (Figures 4A,B), indicating that FBPE may be beneficial to activating the PI3K/AKT signaling pathway. Taken together, it is indicated that FBPE or MET may regulate the expression of inflammatory cytokines in T2DM mice through the PI3K/AKT signaling pathway. Therefore, in the future, it is necessary to conduct experiments at the protein level to further analyze the influence of FBPE on inflammatory cytokine-related signaling pathways in T2DM mice.

#### FBPE Regulates the Gut Microbiota in T2DM Mice

**Figure 5:**
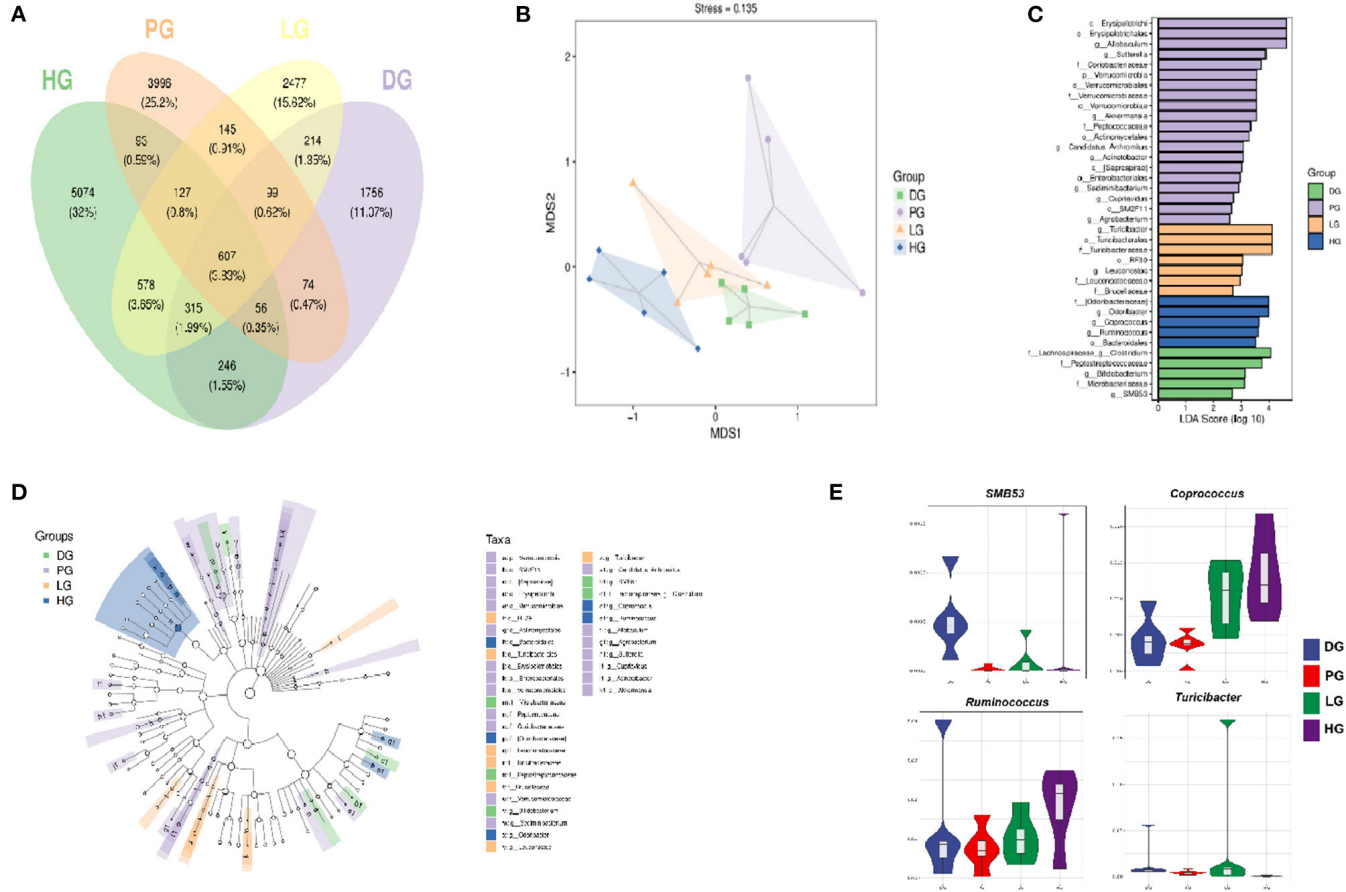
Effects of FBPE or MET on the gut microbiome structure in HFD-STZ induced T2DM mice. **(A)** Venn diagram illustrating the common and unique OTU or taxon between groups. **(B)** unweighted UniFrac NMD analysis showing the clustering of different samples. **(C)** (Green) DG-enriched taxa; (Purple) PG-enriched taxa; (Yellow) LG-enriched taxa; (Blue) HG-enriched taxa. **(D)** LEfSe analysis displaying the most differentially abundant taxons between the DG, PG, LG, and HG. A taxonomic cladogram was obtained from the LEfSe analysis of 16S sequences. **(E)** Highly differentiated OTU at phylum and genus levels in four groups. (*n* = 5).

Following FBPE ingestion, the inflammatory state of T2DM mice was improved along with changes in microbial composition. Here, we performed pyrosequencing of the variable regions V3-V4 of bacterial 16S rRNA genes to evaluate the effect of FBPE on the gut microbiota of C57BL/6J T2DM mice. We detected a total of 2,793,613 sequences, including an average of 103,168 for DG, 112,823 for PG, 128,359 for LG, and 121,251 for HG. The length of these sequences is mostly 420−450 base pairs. (Supplementary Figure 1) The Venn diagram shows the number of common and unique OTUs among 4 groups of mice. As shown in Figure 5A, the qualified sequences (>0.001%) were clustered into 16157 bacterial OTUs. And there are 3,367 OTUs in DG, 5,197 OTUs in PG, 4,562 OTUs in LG, and 7,096 OTUs in HG, respectively. Compared with DG, the number of OTUs in PG, LG, and HG increased by 54.35, 35.49, and 110.75%, respectively. Additionally, we observed that 836 same OTUs were identified in PG and DG, 978 in PG and LG, and 883 in PG and HG. Compared with DG mice, low-dose FBPE and high-dose FBPE administration increased the same OTUs as PG mice.

A rarefaction curve was used to evaluate the diversity of the microbiota; as the increase of the total number of randomly selected sequences, the new ASV or OTUs haven't been discovered. (Supplementary Figure 2A) In addition, the rank abundance curve showed that the community composition of each group is uniform; and the abundance differences of ASV / OTU within the community well small. (Supplementary Figure 2B)

Reports pointed out that T2DM patients' gut microbial structure is related to gut health, and the richness and diversity of the community could be reflected by the diversity index. Among these four indexes, Chao1 and Observed species indexes represent richness while Shannon and Simpson indexes represent diversity. Supplementary Table 5 shows the diversity of the gut microbiota in 4 groups. DG mice performed the lowest diversity among the 4 groups, and the Chao1, Simpson, Shannon, and Observed species indices were increased after MET or FBPE treatment. Particularly, the Chao1 index was remarkably increased (*P* < 0.05) in HG and PG. These data suggested that MET or both doses (1 g/kg BW and 6 g/kg BW) of FBPE had a substantial influence on gut microbiota. Although the diversity was increased but not significantly (Simpson and Shannon), gut microbiota richness was significantly changed (Chao1). The results showed that FBPE intake could alter the diversity of gut microbiota in T2DM mice. The possible reason is that FBPE is rich in polyphenols and most of which are not absorbed in the small intestine, and regulate the composition and function of the colonic microbiota after reaching the colon.

Additionally, Non-metric Multidimensional Scaling (NMDS) analysis based on UniFrac distance was conducted to explore the similarity of gut microbial communities in 4 groups. As shown in Figure 5B, five samples in the DG clustered, and samples in PG and HG were far away from the DG. The results showed that the MET and FBPE treatment induced prominent gut microbial diversification. MET and FBPE consumption at 6 g/kg BW could shift the gut microbiota composition of T2DM mice.

Furthermore, we found that FBPE or MET treatment altered the microbial profile of T2DM mice with some common trends but also some differences. In this paper, MET demonstrated a significant effect (*P* < 0.05) on the species that belonged to the phylum *Actinobacteria, Bacteroidetes, Firmicutes, OD1, Proteobacteria*, and *Verrucomicrobia* (Supplementary Table 6, Figures 5C,D). This is similar to a previous report which showed that MET significantly affects *Firmicutes, Bacteroidetes*, and *Proteobacteria* (20). And the significantly different communities (*P* < 0.05) belonged to *Firmicutes, Proteobacteria, Tenericutes*, and *Bacteroidetes* when supplemented with FBPE in the present study (Supplementary Table 6, Figures 5C,D). At the genus level, the dominant genus in DG were *SMB53* and *Clostridium*, and FBPE treatment could decrease those. In another report, *SMB53* was observed only in Tsumura Suzuki Obese Diabetes (TSOD) mice (54). This is similar to our results. *SMB53* may be important for abnormal metabolism in T2DM. Additionally, the dominant bacteria at the genus level after FBPE administration were *Coprococcus, Ruminococcus*, and *Turicibacter*. As human gut health enhancers, *Coprococcus* has beneficial effects such as fermenting carbohydrates, improving gastrointestinal function, and reducing inflammation. *Ruminococcus* is a butyrate-producing bacterium, which has an anti-inflammatory function and can enhance the intestinal barrier. The study also shows that the relative abundance of *Turicibacter* in HFD induced obese mice was lower than that of mice fed a normal diet (54). The relative abundance of *Turicibacter* in LG mice was significantly increased than that of DG mice in this study. It indicated that the mice's gut environment was more similar to that of mice on a normal diet after FBPE treatment. Recently, potential interactions of polyphenols with gut microbiota have attracted attention (55). Evidence suggests that these interactions may modulate chronic diseases such as improved insulin sensitivity (56, 57). And reports proved that phenolic compounds may promote beneficial actions of probiotics (58). It indicated that the gut microbiota structure modulation effects of FBPE in T2DM mice could be associated with the polyphenols.

A study revealed that alterations in gut microbiota composition are related to low-grade inflammation, obesity, and T2DM in mice (59). Hence, we analyzed the correlation between inflammatory factors and gut microbiota communities. As shown in Supplementary Table 7, some key communities associated with TNF-α and TGF-β were observed, *Candidatus Arthromitus* and *SMB53* showed positive correlations to TNF-α, *Coprococcus, Ruminocossus*, and *Odoribacteraceae* reported negative correlations to TGF-β.

## Conclusion

In conclusion, FBPE contained 8 phenolic compounds and possessed antioxidant activity. *In vivo* animal experiments showed that FBPE could attenuate BW, decrease FBG, attenuate oxidative injury, cut down the lipid accumulation, and lessen inflammation in T2DM mice. In addition, FBPE ingestion activated the PI3K/AKT signaling pathway and repaired liver and epididymal fat damage induced by HFD and STZ. Furthermore, FBPE can modulate diabetes-induced gut microbiota disturbances, which could be related to the polyphenols contained in FBPE. This study could provide a theoretical preference for the hypoglycemic mechanism of FBPE, which could be used as potential anti-diabetic medicine.

## Data Availability Statement

The original contributions presented in the study are included in the article/Supplementary Material, further inquiries can be directed to the corresponding author/s.

## Ethics Statement

The animal study was reviewed and approved by Animal Ethics Committee of Northwest University.

## Author Contributions

JZ: investigation, data curation, and writing-original draft. HZ: investigation and writing-reviewing and editing. SG, QW, and NC: methodology. NB: resources. WC: project administration, funding acquisition, and writing-reviewing and editing. All authors contributed to the article and approved the submitted version.

## Funding

This work was financially supported by the National Natural Science Foundation of China (31871876), the Shaanxi Science and Technology Project (2022FP-12 and 2022FP-14), and the Shaanxi High-Level Talent Special Support Plan (TZ0389).

## Conflict of Interest

The authors declare that the research was conducted in the absence of any commercial or financial relationships that could be construed as a potential conflict of interest.

## Publisher's Note

All claims expressed in this article are solely those of the authors and do not necessarily represent those of their affiliated organizations, or those of the publisher, the editors and the reviewers. Any product that may be evaluated in this article, or claim that may be made by its manufacturer, is not guaranteed or endorsed by the publisher.

## Abbreviations

AKT: protein kinase B
ALP: alkaline phosphatase
ALT: cereal third transaminase
AST: aspartate transaminase
BW: body weight
^13^C-NMR: carbon nuclear magnetic resonance spectrum
DM: diabetes mellitus
DPPH, 1: 1-Diphenyl-2-picrylhydrazyl radical
FBG: fasting blood glucose
FBP: *Fagopyrum esculentum* Moench. bee pollen
FBPE: *F. esculentum* bee pollen extract
GSH-Px: glutathione peroxidase
GSK-3β: glycogen synthesis kinase-3β
H&E: hematoxylin and eosin
HDL-C: high-density lipoprotein-cholesterol
HFD: high-fat-diet
^1^H-NMR: hydrogen nuclear magnetic resonance spectrum
HOMA-IR: homeostasis model assessment of insulin resistance
HOMA-β: homeostasis model assessment-β
HPLC: high performance liquid chromatography
IL-2: interleukin-2
IL-6: interleukin-6
ISI: insulin sensitivity index
LDL-C: low-density lipoprotein-cholesterol
MDA: malondialdehyde
MET: metformin
MS: mass spectrometry
NaCMC: carboxymethylcellulose sodium solution
NMDS: Nonmetric Multidimensional Scaling
PCR: polymerase chain reaction
PI3K: phosphatidylinositol 3-kinase
ROS: reactive oxygen species
SOD: superoxide dismutase
STZ: streptozocin
T1DM: type I diabetes mellitus
TC: total cholesterol
TFC: total flavonoids content
TG: triglyceride
TGF-β: transforming growth factor-β
TLC: thin-layer chromatography
TNF-α: tumor necrosis factor-α
TP: total protein
TPC: total phenolic content
UV: ultraviolet spectrum.
