# Supplementary material for: Protective Mechanism of Fagopyrum esculentum Moench. Bee Pollen EtOH Extract Against Type II Diabetes in a High-Fat Diet/Streptozocin-Induced C57BL/6J Mice
Source: Front Nutr. 2022 Jun 30;9:925351. doi: 10.3389/fnut.2022.925351 (PMC9280863; doi:10.3389/fnut.2022.925351)
Supplement: Supplementary file 1 [file Data_Sheet_1.docx]

Supplementary Material

# Supplementary Figures and Tables

## Supplementary Figures


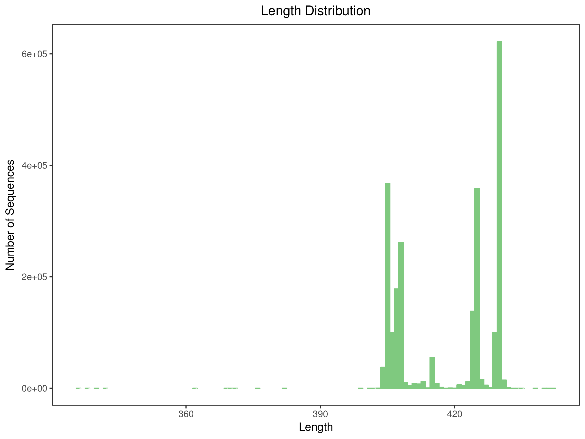


**Supplementary Figure 1.** DNA sequencing number and length.


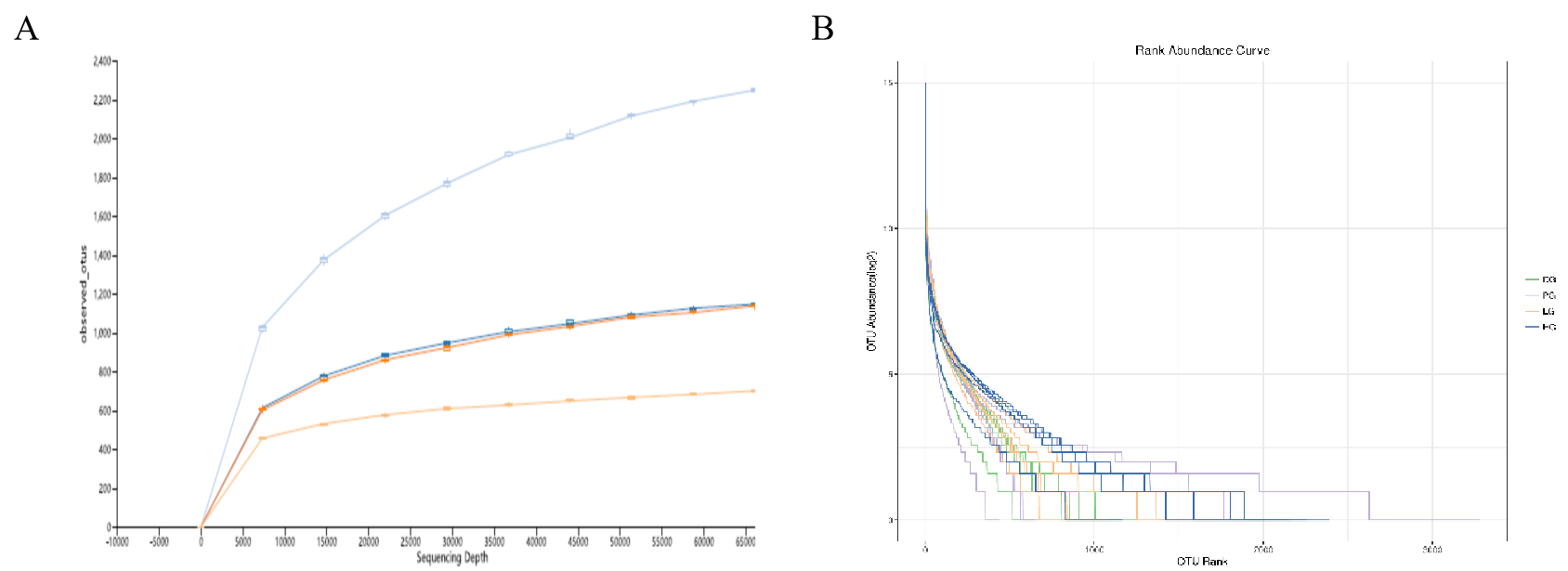


**Supplementary Figure 2.** Rarefaction curve (A) and Rank Abundance curve (B) of OTU number of colon samples in four groups

DG: (STZ, Model group); PG: (STZ, MET 150 mg/kg); LG: (STZ, 1 g/kg FBPE); HG: (STZ, 6 g/kg FBPE)

## Supplementary Tables

**Supplementary Table 1** The primer sequences

| **Gene** | **Primers position** | **Primers sequences(5’→3’)** |
| --- | --- | --- |
| TNF-α | Forward | AGCCGATGGGTTGTACCTTG |
|  | Reverse | AGTACTTGGGCAGATTGACCTC |
| TGF-β | Forward | GCCTTAGTTTGGACAGGATCTG |
|  | Reverse | CTCCCGTGGCTTCTAGTGC |
| PI3K | Forward | GAGGATTTGCCCCACCATGA |
|  | Reverse | ACGCAATGTTTGACTTCGCC |
| AKt | Forward | GAGAACCGTGTCCTGCAGAA |
|  | Reverse | GTTCTCCAGCTTGAGGTCCC |
| IL-2 | Forward | CATCTTCAGTGCCTGGAA |
|  | Reverse | GTGGTGGAATTTCTGAGGA |
| IL-6 | Forward | GCCACTGCCTTCCCTACTTC |
|  | Reverse | TCTGACAGTGCATCATCGCT |
| GAPDH | Forward | GGTGGACCTCATGGCCTACA |
|  | Reverse | CTCTCTTGCTCTCAGTATCCTTGCT |

**Supplementary Table 2** The results of TPC, TFC and antioxidant of FBPE in vitro

| **TPC/(g GAE/kg)** | **TFC/(g RE/kg)** | **DPPH scavenging activity/IC_50_ (mg/mL)** | **Ferrous ion-chelating activity/(mg Na2EDTA/g)** |
| --- | --- | --- | --- |
| 18.59±1.39 | 16.35±0.09 | 11.01±0.02 | 32.84±1.49 |

Results presented in the table are expressed as mean ± standard deviation (SD) for 3 replications.

**Supplementary Table 3** The content of 8 phenolic compounds of FBPE

| **Peak** | **chlorogenic acid** | **caffeic acid** | **resveratrol** | **catechin** | **luteolin** | **rutin** | **kaempferol** | **quercetin** |
| --- | --- | --- | --- | --- | --- | --- | --- | --- |
| Content (g/kg) | 1.45 | 3.47 | 5.25 | 2.40 | 9.46 | 1.45 | 3.67 | 2.66 |

**Supplementary Table 4** Effects of FBPE or MET on biochemical parameters in T2DM mice

|  | **DG** | **PG** | **LG** | **HG** |
| --- | --- | --- | --- | --- |
| Serum |  |  |  |  |
| TC (mmol/L) | 6.94±0.91 | 6.53±1.13 | 6.85±2.31 | 5.77±1.33 |
| TG (mmol/L) | 1.36±0.27^b^ | 1.00±0.25^ab^ | 1.00±0.25^ab^ | 0.87±0.24^a^ |
| HDL-C(mmol/L) | 3.52±0.58 | 3.84±0.39 | 3.83±0.29 | 3.54±0.62 |
| LDL-C(mmol/L) | 1.04±0.09^b^ | 0.83±0.22^ab^ | 0.78±0.17^ab^ | 0.59±0.18^a^ |
| ALB(g/L) | 22.05±4.38 | 21.09±5.33 | 23.48±4.41 | 21.74±6.62 |
| AKP(/100mL) | 13.29±2.08 | 11.55±1.29 | 11.85±2.80 | 14.16±3.12 |
| ALT | 58.92±12.25^b^ | 51.09±9.97^ab^ | 35.92±0.86^a^ | 32.69±7.36^a^ |
| AST | 67.88±2.58^b^ | 56.14±4.23^a^ | 74.82±5.52^b^ | 54.09±7.28^a^ |
| Liver |  |  |  |  |
| TC(mmol/gprot) | 0.12±0.03^b^ | 0.11±0.02^ab^ | 0.08±0.02^ab^ | 0.06±0.00^a^ |
| TG(mmol/gprot) | 0.39±0.05^b^ | 0.25±0.03^a^ | 0.20±0.03^a^ | 0.27±0.10^a^ |
| LG(mg/g liver) | 6.44±0.78^a^ | 6.81±1.31^a^ | 12.05±1.42^b^ | 6.29±0.58^a^ |
| TP(gprot/L) | 7.36±1.01^a^ | 9.59±0.81^a^ | 9.34±1.60^a^ | 8.97±1.93^a^ |
| MDA(nmol/mgprot) | 3.99±0.63^b^ | 2.07±0.78^b^ | 1.24±0.22^ab^ | 0.69±0.06^a^ |
| SOD(U/mgprot) | 344.17±31.82^a^ | 357.39±7.79^a^ | 340.37±26.46^a^ | 444.99±24.64^b^ |
| GSH-Px(U/mgprot) | 156.50±5.19^a^ | 197.91±13.93^b^ | 209.44±8.56^b^ | 193.54±8.30a^b^ |

Different letters means significantly different, *P* < 0.05.

**Supplementary Table 5** Effects of FBPE on the alpha diversity index of gut microbiota

|  | **Chao1** | **Simpson** | **Shannon** | **Observed species** |
| --- | --- | --- | --- | --- |
| DG | 1297±101^a^ | 0.9551±0.0235^a^ | 6.69±0.44^a^ | 1193±102^a^ |
| LG | 1542±456^ab^ | 0.9730±0.0168^a^ | 7.15±0.61^a^ | 1490±416^a^ |
| HG | 2279±422^bc^ | 0.9665±0.0120^a^ | 7.78±0.38^a^ | 2068±487^ab^ |
| PG | 2755±363^c^ | 0.9797±0.0124^a^ | 8.16±0.65^a^ | 2638±358^b^ |

Different letters means significantly different, *P* < 0.05.

**Supplementary Table 6** The significant differently species determined based on the LEfSe method using the nonparametric factorial Kruskal-Wallis rank sum test.

| **Group** | **Taxa** | **Relative Abundance** | **LDA score** | **P-value** |
| --- | --- | --- | --- | --- |
| DG | Actinobacteria.Actinobacteria.Actinomycetales.Microbacteriaceae | 2.29 | 3.13 | 0.01 |
|  | Actinobacteria.Actinobacteria.Bifidobacteriales.Bifidobacteriaceae.Bifidobacterium | 3.55 | 3.14 | 0.04 |
|  | Firmicutes.Clostridia.Clostridiales.Clostridiaceae.SMB53 | 2.75 | 2.69 | 0.03 |
|  | Firmicutes.Clostridia.Clostridiales.Lachnospiraceae.Clostridium | 4.38 | 4.07 | 0.03 |
|  | Firmicutes.Clostridia.Clostridiales.Peptostreptococcaceae | 4.19 | 3.76 | 0.02 |
| PG | Actinobacteria.Actinobacteria.Actinomycetales | 2.52 | 3.30 | 0.03 |
|  | Actinobacteria.Coriobacteriia.Coriobacteriales.Coriobacteriaceae | 4.06 | 3.73 | 0.02 |
|  | Bacteroidetes._Saprospirae_ | 3.34 | 3.02 | 0.04 |
|  | Bacteroidetes._Saprospirae_._Saprospirales_.Chitinophagaceae.Sediminibacterium | 3.23 | 2.90 | 0.04 |
|  | Firmicutes.Clostridia.Clostridiales.Clostridiaceae.Candidatus_Arthromitus | 3.08 | 3.08 | 0.04 |
|  | Firmicutes.Clostridia.Clostridiales.Peptococcaceae | 2.26 | 3.34 | 0.04 |
|  | Firmicutes.Erysipelotrichi | 5.08 | 4.64 | 0.03 |
|  | Firmicutes.Erysipelotrichi.Erysipelotrichales | 5.08 | 4.64 | 0.03 |
|  | Firmicutes.Erysipelotrichi.Erysipelotrichales.Erysipelotrichaceae.Allobaculum | 5.07 | 4.64 | 0.03 |
|  | OD1.SM2F11 | 2.24 | 2.66 | 0.02 |
|  | Proteobacteria.Alphaproteobacteria.Rhizobiales.Rhizobiaceae.Agrobacterium | 2.66 | 2.59 | 0.04 |
|  | Proteobacteria.Betaproteobacteria.Burkholderiales.Alcaligenaceae.Sutterella | 4.15 | 3.89 | 0.02 |
|  | Proteobacteria.Betaproteobacteria.Burkholderiales.Oxalobacteraceae.Cupriavidus | 2.97 | 2.74 | 0.01 |
|  | Proteobacteria.Gammaproteobacteria.Enterobacteriales | 3.26 | 2.96 | 0.05 |
|  | Proteobacteria.Gammaproteobacteria.Pseudomonadales.Moraxellaceae.Acinetobacter | 3.52 | 3.08 | 0.04 |
|  | Verrucomicrobia | 3.83 | 3.56 | 0.00 |
|  | Verrucomicrobia.Verrucomicrobiae | 3.82 | 3.55 | 0.00 |
|  | Verrucomicrobia.Verrucomicrobiae.Verrucomicrobiales | 3.82 | 3.55 | 0.00 |
|  | Verrucomicrobia.Verrucomicrobiae.Verrucomicrobiales.Verrucomicrobiaceae | 3.82 | 3.55 | 0.00 |
|  | Verrucomicrobia.Verrucomicrobiae.Verrucomicrobiales.Verrucomicrobiaceae.Akkermansia | 3.81 | 3.55 | 0.00 |
| LG | Firmicutes.Bacilli.Lactobacillales.Leuconostocaceae | 1.90 | 2.96 | 0.03 |
|  | Firmicutes.Bacilli.Lactobacillales.Leuconostocaceae.Leuconostoc | 1.90 | 3.03 | 0.03 |
|  | Firmicutes.Bacilli.Turicibacterales | 4.58 | 4.12 | 0.02 |
|  | Firmicutes.Bacilli.Turicibacterales.Turicibacteraceae | 4.58 | 4.12 | 0.02 |
|  | Firmicutes.Bacilli.Turicibacterales.Turicibacteraceae.Turicibacter | 4.58 | 4.12 | 0.02 |
|  | Proteobacteria.Alphaproteobacteria.Rhizobiales.Brucellaceae | 2.98 | 2.72 | 0.05 |
|  | Tenericutes.Mollicutes.RF39 | 2.62 | 3.06 | 0.01 |
| HG | Bacteroidetes.Bacteroidia.Bacteroidales | 3.92 | 3.52 | 0.02 |
|  | Bacteroidetes.Bacteroidia.Bacteroidales._Odoribacteraceae_ | 4.33 | 3.98 | 0.02 |
|  | Bacteroidetes.Bacteroidia.Bacteroidales._Odoribacteraceae_.Odoribacter | 4.32 | 3.98 | 0.02 |
|  | Firmicutes.Clostridia.Clostridiales.Lachnospiraceae.Coprococcus | 4.12 | 3.64 | 0.01 |
|  | Firmicutes.Clostridia.Clostridiales.Ruminococcaceae.Ruminococcus | 4.13 | 3.62 | 0.04 |

DG: (STZ, Model group); PG: (STZ, MET 150 mg/kg); LG: (STZ, 1 g/kg FBPE); HG: (STZ, 6 g/kg FBPE)

**Supplementary Table 7** Correlation analysis of inflammatory factors and intestinal significantly different bacteria

|  | **IL-2** | **IL-6** | **TNF-α** | **TGF-β** | **P13K** | **Akt** |
| --- | --- | --- | --- | --- | --- | --- |
|  | **Spearman’ correlation R** | | | | | |
| Actinobacteria.Actinobacteria.Actinomycetales | 0.200 | 0.115 | 0.056 | 0.266 | -0.594* | -0.527 |
| Actinobacteria.Actinobacteria.Actinomycetales.Microbacteriaceae | 0.059 | 0.274 | 0.374 | 0.762** | -0.109 | -0.115 |
| Actinobacteria.Actinobacteria.Bifidobacteriales.Bifidobacteriaceae.Bifidobacterium | -0.136 | 0.141 | 0.359 | 0.592* | -0.444 | -0.229 |
| Actinobacteria.Coriobacteriia.Coriobacteriales.Coriobacteriaceae | -0.133 | 0.442 | 0.545 | 0.769** | 0.273 | -0.145 |
| Bacteroidetes.Bacteroidia.Bacteroidales | 0.067 | -0.333 | -0.503 | -0.273 | 0.056 | 0.427 |
| Bacteroidetes.Bacteroidia.Bacteroidales._Odoribacteraceae_ | -0.150 | -0.176 | -0.301 | -0.629* | 0.133 | -0.018 |
| Bacteroidetes.Bacteroidia.Bacteroidales._Odoribacteraceae_.Odoribacter | 0.067 | -0.127 | -0.203 | -.552 | 0.189 | -0.118 |
| Bacteroidetes._Saprospirae_ | 0.050 | 0.115 | 0.280 | 0.639* | -0.007 | 0.118 |
| Bacteroidetes._Saprospirae_._Saprospirales_.Chitinophagaceae.Sediminibacterium | 0.267 | 0.261 | 0.298 | 0.613* | -0.011 | 0.036 |
| Firmicutes.Bacilli.Lactobacillales.Leuconostocaceae | 0.166 | -0.330 | -0.004 | -0.064 | -0.860** | -0.594 |
| Firmicutes.Bacilli.Lactobacillales.Leuconostocaceae.Leuconostoc | 0.166 | -0.330 | -0.004 | -0.064 | -0.860** | -0.594 |
| Firmicutes.Bacilli.Turicibacterales | 0.209 | 0.043 | 0.256 | -0.494 | -0.578* | -0.433 |
| Firmicutes.Bacilli.Turicibacterales.Turicibacteraceae | 0.209 | 0.043 | 0.256 | -0.494 | -0.578* | -0.433 |
| Firmicutes.Bacilli.Turicibacterales.Turicibacteraceae.Turicibacter | 0.209 | 0.043 | 0.256 | -0.494 | -0.578* | -0.433 |
| Firmicutes.Clostridia.Clostridiales.Clostridiaceae.Candidatus_Arthromitus | 0.201 | 0.369 | 0.811** | 0.632* | -0.460 | -0.402 |
| Firmicutes.Clostridia.Clostridiales.Clostridiaceae.SMB53 | 0.183 | 0.493 | 0.734** | 0.569 | -0.606* | -0.740** |
| Firmicutes.Clostridia.Clostridiales.Lachnospiraceae.Clostridium | 0.383 | 0.285 | 0.098 | 0.126 | -0.692* | -0.800* |
| Firmicutes.Clostridia.Clostridiales.Lachnospiraceae.Coprococcus | -0.233 | -0.309 | -0.462 | -0.734** | 0.217 | 0.236 |
| Firmicutes.Clostridia.Clostridiales.Peptococcaceae | -0.151 | 0.153 | -0.143 | 0.000 | 0.714** | 0.825** |
| Firmicutes.Clostridia.Clostridiales.Peptostreptococcaceae | 0.170 | 0.181 | 0.253 | 0.057 | -0.879** | -0.881** |
| Firmicutes.Clostridia.Clostridiales.Ruminococcaceae.Ruminococcus | -0.367 | -0.261 | -0.462 | -0.748** | 0.231 | 0.282 |
| Firmicutes.Erysipelotrichi | 0.100 | 0.503 | 0.427 | 0.559 | -0.329 | -0.364 |
| Firmicutes.Erysipelotrichi.Erysipelotrichales | 0.100 | 0.503 | 0.427 | 0.559 | -0.329 | -0.364 |
| Firmicutes.Erysipelotrichi.Erysipelotrichales.Erysipelotrichaceae.Allobaculum | 0.033 | 0.297 | 0.448 | 0.490 | -0.490 | -0.291 |
| OD1.SM2F11 | 0.101 | -0.363 | -0.445 | -0.022 | 0.273 | 0.671* |
| Proteobacteria.Alphaproteobacteria.Rhizobiales.Brucellaceae | -0.008 | 0.413 | 0.490 | 0.771** | 0.144 | 0.023 |
| Proteobacteria.Alphaproteobacteria.Rhizobiales.Rhizobiaceae.Agrobacterium | 0.114 | 0.119 | 0.090 | 0.647* | 0.136 | 0.104 |
| Proteobacteria.Betaproteobacteria.Burkholderiales.Alcaligenaceae.Sutterella | 0.167 | 0.006 | -0.112 | 0.252 | 0.007 | 0.355 |
| Proteobacteria.Betaproteobacteria.Burkholderiales.Oxalobacteraceae.Cupriavidus | 0.343 | 0.055 | 0.537 | 0.498 | -0.418 | -0.123 |
| Proteobacteria.Gammaproteobacteria.Enterobacteriales | 0.368 | 0.213 | 0.014 | 0.109 | -0.608* | -0.562 |
| Proteobacteria.Gammaproteobacteria.Pseudomonadales.Moraxellaceae.Acinetobacter | 0.075 | 0.238 | 0.239 | 0.628* | -0.098 | -0.018 |
| Tenericutes.Mollicutes.RF39 | 0.044 | -0.119 | -0.403 | 0.033 | 0.618* | 0.597 |
| Verrucomicrobia | -0.035 | -0.400 | -0.289 | -0.065 | -0.177 | 0.443 |
| Verrucomicrobia.Verrucomicrobiae | 0.085 | -0.141 | -0.092 | 0.437 | 0.011 | 0.230 |
| Verrucomicrobia.Verrucomicrobiae.Verrucomicrobiales | 0.085 | -0.141 | -0.092 | 0.437 | 0.011 | 0.230 |
| Verrucomicrobia.Verrucomicrobiae.Verrucomicrobiales.Verrucomicrobiaceae | 0.085 | -0.141 | -0.092 | 0.437 | 0.011 | 0.230 |
| Verrucomicrobia.Verrucomicrobiae.Verrucomicrobiales.Verrucomicrobiaceae.Akkermansia | -0.034 | 0.067 | -0.152 | 0.377 | 0.018 | 0.290 |

DG (STZ, Model group); PG: (STZ, MET 150 mg/kg); LG: (STZ, 1 g/kg FBPE); HG: (STZ, 6 g/kg FBPE)

** Spearman’ correlation is significantly different at a level of 0.01; *Spearman’ correlation is significantly different at a level of 0.05.
